# *Drosophila* Pkaap regulates Rab4/Rab11-dependent traffic and Rab11 exocytosis of innate immune cargo

**DOI:** 10.1242/bio.016642

**Published:** 2016-05-17

**Authors:** Alexandra Sorvina, Tetyana Shandala, Douglas A. Brooks

**Affiliations:** Sansom Institute for Health Research, University of South Australia, Adelaide, South Australia 5001, Australia

**Keywords:** *Drosophila*, Innate immunity, Endosomes, Rab4, Rab11, Pkaap, Antimicrobial peptide Drosomycin

## Abstract

The secretion of immune-mediators is a critical step in the host innate immune response to pathogen invasion, and Rab GTPases have an important role in the regulation of this process. Rab4/Rab11 recycling endosomes are involved in the sorting of immune-mediators into specialist Rab11 vesicles that can traffic this cargo to the plasma membrane; however, how this sequential delivery process is regulated has yet to be fully defined. Here, we report that *Drosophila* Pkaap, an orthologue of the human dual-specific A-kinase-anchoring protein 2 or D-AKAP2 (also called AKAP10), appeared to have a nucleotide-dependent localisation to Rab4 and Rab11 endosomes. RNAi silencing of *pkaap* altered Rab4/Rab11 recycling endosome morphology, suggesting that Pkaap functions in cargo sorting and delivery in the secretory pathway. The depletion of *pkaap* also had a direct effect on Rab11 vesicle exocytosis and the secretion of the antimicrobial peptide Drosomycin at the plasma membrane. We propose that Pkaap has a dual role in antimicrobial peptide traffic and exocytosis, making it an essential component for the secretion of inflammatory mediators and the defence of the host against pathogens.

## Abstract (precis)

**Summary:** Pkaap has a dual role in antimicrobial peptide trafficking and exocytosis, making it an essential component for the secretion of inflammatory mediators and the defence of the host against pathogens.

## INTRODUCTION

The innate immune system enables a coordinated host response to microorganism invasion, resulting in the secretion of immune-mediators, which can either induce inflammation or kill pathogens (Beisswenger and Bals, 2005; Cederlund et al., 2011; Jenssen et al., 2006; Lai and Gallo, 2009; Stow and Murray, 2013). However, excessive or uncontrolled secretion of immune-mediators can be associated with important pathological states, including rheumatoid arthritis, diabetes, Alzheimer's, multiple sclerosis, cancer and cardiovascular disease (Charo and Ransohoff, 2006; Compston and Coles, 2002; Grateau, 2006; Hansson and Libby, 2006; Hotamisligil, 2006; McInnes and Schett, 2007). The critical role of innate immunity in host defence and inflammation has warranted studies on the molecular mechanisms involved in generating an effective immune response and for the secretion of specific immune cargo.

The Toll-like receptors recognise pathogens and initiate an innate immune response (Barton and Kagan, 2009). This pathogen recognition system at the cell surface of immune cells can trigger different intracellular signalling cascades, including the Toll-interleukin 1 pathway or the immune-deficiency-tumour necrosis factor alpha (TNFα) pathway, which results in the synthesis of immune-mediators (Lemaitre, 2004). Cytokines and antimicrobial peptides that are synthesised in response to this immune stimulation are subsequently delivered to the endosomal system for processing and packaging into specialist exocytic vesicles (Bonifacino and Glick, 2004). Small Rab GTPases are critically involved in the regulation of this sequential transport process, with cargo sorting in Rab4/Rab11 recycling endosomes (Ang et al., 2004), and traffic to the plasma membrane in Rab11 vesicles (Chen et al., 1998; Damiani et al., 2004; Li et al., 2008; Mohrmann et al., 2002; Shandala et al., 2011). Rab11-mediated exocytosis has been reported for various cytokines, including TNFα (Murray et al., 2005; Reefman et al., 2010), interferon gamma (Reefman et al., 2010), interleukin 10 (Stanley et al., 2012) in mammals, and the antimicrobial peptide Drosomycin in *Drosophila* (Shandala et al., 2011). While the immune signalling cascades as well as the synthesis and delivery of cytokines and antimicrobial peptides have been well characterised, the regulatory mechanisms for Rab11-mediated exocytosis and release of innate immune-mediators have yet to be fully defined.

GTPase activating proteins (GAPs) and guanine nucleotide exchange factors (GEFs) are known to regulate Rab GTPase activity, but these regulatory elements have yet to be defined for innate immune cargo delivery and secretion at the plasma membrane (Bos et al., 2007; Csépányi-Kömi et al., 2012; Liao et al., 2008). GAPs inactivate Rab GTPases by stimulating the hydrolysis of guanosine triphosphate (GTP) to guanosine diphosphate (GDP; Scheffzek and Ahmadian, 2005; Zurita et al., 2010), whereas GEFs facilitate their activation by exchanging GDP for GTP (Bos et al., 2007; Liao et al., 2008). Multiple GAPs and GEFs have been reported to be involved in Rab11 nucleotide exchange. For example, TBC1D15 exhibits GAP activity against Rab11, but can also affect the activity of Rab7 (Zhang et al., 2005). Similarly, TBC1D11/GAPCenA exhibits GAP activity to both Rab4 and Rab11 GTPases (Fuchs et al., 2007). The *Drosophila* protein Evi5 acts as a GAP for Rab11 to regulate the migration of border cells (Dabbeekeh et al., 2007; Laflamme et al., 2012). In contrast, the vesicular transport of Rhodospin to the rhabdomere membrane involves Rab11 activation by Calmodulin-binding protein related to a Rab3 GDP/GTP exchange protein (also called Crag) that acts as a GEF in *Drosophila* photoreceptors (Xiong et al., 2012). The dual-specific A-kinase-anchoring protein 2 (also called D-AKAP2 or AKAP10) appears to have GEF activity towards Rab11 in humans (Eggers et al., 2009). Two highly conserved regulatory G protein signalling (RGS) domains in D-AKAP2, referred to as RGS1 and RGS2, are sufficient to bind simultaneously to both Rab4 and Rab11 (Fukuda et al., 2008), and this is used in the regulation of transferrin receptor recycling in HeLa cells (Eggers et al., 2009). D-AKAP2 preferentially binds to inactive GDP-locked *Rab11^S25N^* and active GTP-locked *Rab4^Q67L^*, and therefore D-AKAP2 may exhibit GEF activity towards Rab11 and GAP activity towards Rab4 (Eggers et al., 2009). Consequently, proteins involved in the regulation of Rab11 can be very selective and function preferentially on this GTPase, while others are promiscuous and regulate multiple Rab proteins, making it difficult to define the precise mechanism controlling innate immune secretion.

Little is known about the GEF and GAP proteins that are involved in regulating Rab11 function during an immune response and how this impacts on the secretion of immune-mediators. Here, we have investigated *Drosophil*a Pkaap, an orthologue of human D-AKAP2, as a candidate for the regulation of Rab4/Rab11 sorting and Rab11 exocytosis of innate immune cargo. The response to *pkaap^RNAi^* was studied in live mode in the fat body, due to the large cell size (high DNA ploidy) and proportionally enlarged endosome/exocytic compartments, which facilitated the visualisation of molecular events during an immune response. The specific focus on *Drosophila* Pkaap was based on previous evidence for D-AKAP2 appearing to have a role in exocytosis (Eggers et al., 2009) and the immune response (Kim et al., 2011).

## RESULTS

### *pkaap* depletion increased *Drosophila* susceptibility to acute bacterial infection

**Fig. 1.**
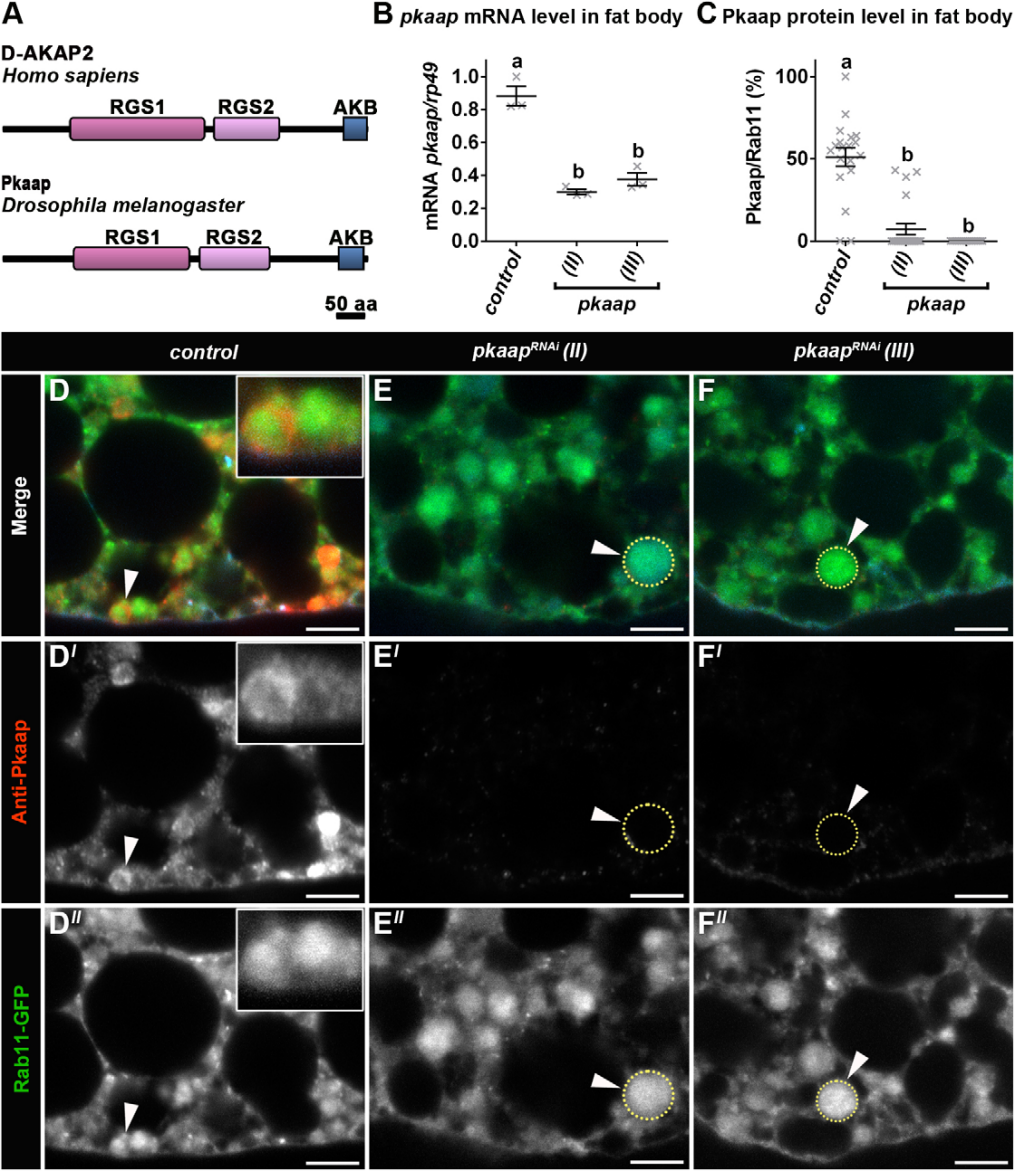
**Expression of *CG-GAL4>UAS-pkaap* in fat body tissue.** (A) A schematic diagram showing the domain structure of D-AKAP2 and Pkaap proteins. D-AKAP2 and Pkaap proteins contain regulator of G protein signalling (RGS) domains and PKA-binding (AKB) domain. (B) The expression of *pkaap* mRNA was characterised by quantitative real-time PCR in fat body tissue from the following genotypes: control, *pkaap^RNAi chromosome II^* and *pkaap^RNAi chromosome III^*. mRNA levels were normalised against *rp49* mRNA levels. Three independent sets of samples were analysed from late third larval instar (−4 h puparium formation). One-way ANOVA and Tukey's multiple comparison test showed significant differences between the means for the genotypes (depicted by different letters on the bars, *P*<0.0001). Data presented as mean±s.e.m. (C) Percentage of Pkaap colocation with Rab11 vesicles in the fat body cells from the following genotypes: control, *pkaap^RNAi chromosome II^* and *pkaap^RNAi chromosome III^*. One-way ANOVA and Tukey's multiple comparison test showed significant differences between the means for the genotypes (depicted by different letters on the bars, *P*<0.0001). (D-F) Confocal micrographs showing localisation of Pkaap detected with an anti-Pkaap antibody (red in D-F; greyscale in D′-F′) in relation to Rab11-GFP vesicles (green in D-F; greyscale in D″-F″) in (D) control, (E) *pkaap^RNAi chromosome II^* and (F) *pkaap^RNAi chromosome III^* fat bodies. The plasma membrane was detected with Alexa Fluor^®^ 568 Phalloidin (cyan in D-F). Data is representative of at least ten independent replicates. Scale bar: 5 μm.

The *pkaap* gene (CG4132) encodes a 662 amino acid protein that is 27% identical (152/571) to human D-AKAP2. Pkaap and D-AKAP2 share two tandem conserved regulatory G protein signalling domains and a protein kinase A (PKA)-binding domain (Fig. 1A). Pkaap RGS1 and RGS2 domains displayed respectively 22% and 34% sequence identity with those of D-AKAP2. RNAi silencing of *pkaap* significantly reduced mRNA levels of *pkaap* (*P*<0.0001; Fig. 1B) and the amount of Pkaap protein detected in *Drosophila* fat body tissue (Fig. 1C,E,E′,F,F′), when compared to control larval tissue (Fig. 1D,D′). There was a significant reduction in the survival of *pkaap^RNAi^ Drosophila* larvae, when compared to control third instar larvae (*P*<0.05). The viability was 91.7±1.5% for control larvae, compared to 82.0±1.9% for *pkaap^RNAi chromosome II^* and 83.1±1.9% for *pkaap^RNAi^* ^*chromosome III*^ transgenic larvae. When *Drosophila* early third instar larvae were infected with a combination of Gram-positive *Micrococcus luteus* and Gram-negative *Escherichia coli*, there was significantly reduced survival for *pkaap^RNAi^* transgenic larvae compared to control larvae (*P*<0.05). The viability was 84.6±2.3% for control larvae, compared to 80.0±2.3% for *pkaap^RNAi^* ^*chromosome II*^ and 63.3±5.4% for *pkaap^RNAi chromosome III^* transgenic larvae. The reduced survival of *pkaap^RNAi^* transgenic larvae compared to control larvae, either without or with bacterial challenge, might be attributed to a potential problem in immune function.

### *pkaap* depletion resulted in altered Rab11 vesicle morphology and exocytosis

**Fig. 2.**
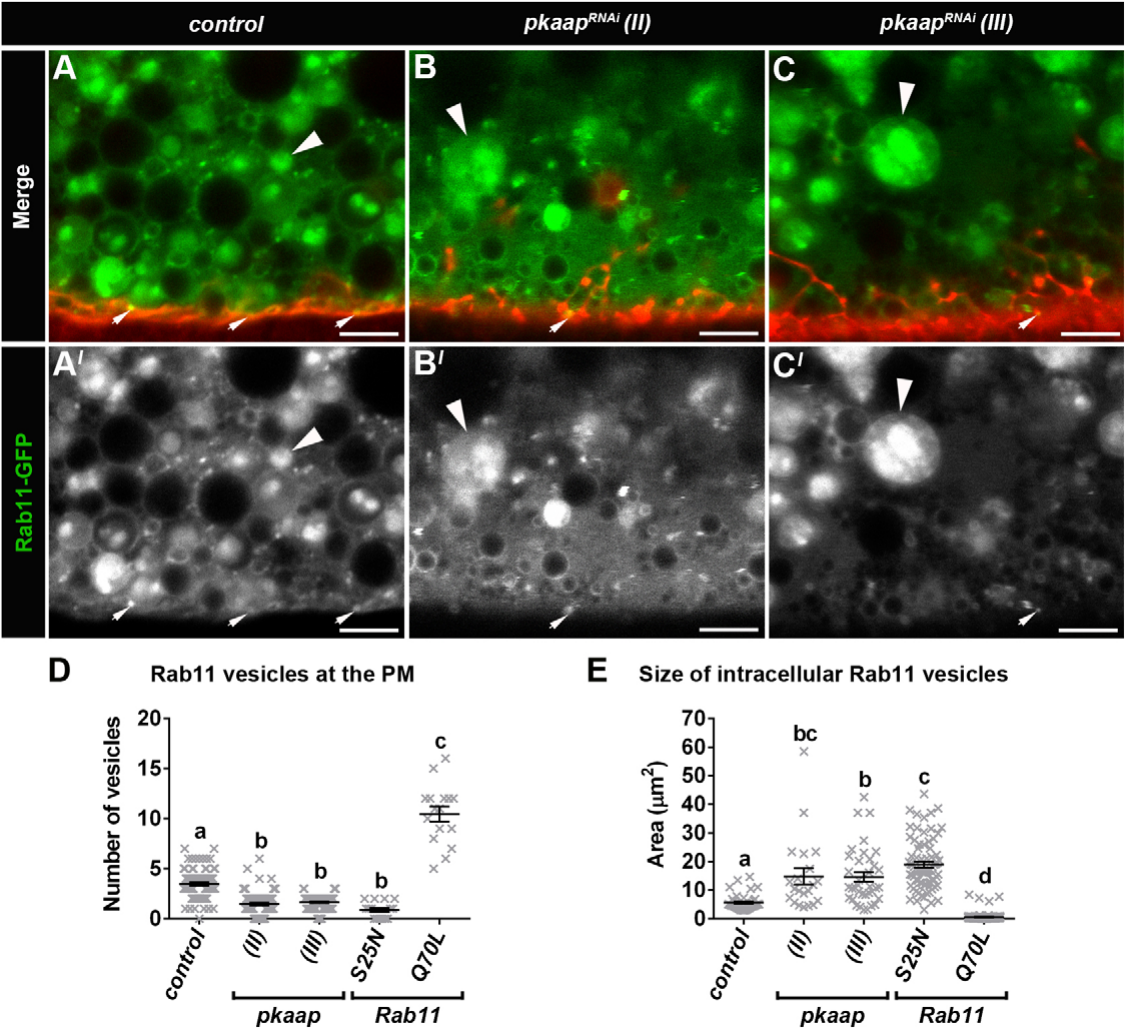
***pkaap* depletion resulted in a decreased number of Rab11 vesicles at the plasma membrane and changed the morphology of intracellular Rab11 endosomes.** (A-C) Confocal micrographs of cross-section through the fat body cells showing Rab11-GFP vesicles (green in A-C; greyscale in A′-C′) with the plasma membrane outlined by CellMask™ Deep Red (red in A-C). Arrows depict small ≤1 μm^2^ Rab11 vesicles at the plasma membrane. Arrowheads depict large Rab11-GFP endosomes with area ≥3 μm^2^. Representative larval fat body cells were from the following genotypes: (A) control, (B) *pkaap^RNAi chromosome II^* and (C) *pkaap^RNAi chromosome III^*. Scale bar: 5 μm. Histograms showing (D) comparative analysis of the number of small ≤1 μm^2^ Rab11 vesicles at the plasma membrane, and (E) size of intracellular Rab11 endosomes with area ≥3 μm^2^. One-way ANOVA and Tukey's multiple comparison test showed significant differences between the means for the genotypes (depicted by different letters on the bars, *P*<0.0001). Data presented as mean±s.e.m. PM, plasma membrane.

In control *Drosophila* fat body tissue from third larval instars, Pkaap protein colocated with Rab11 vesicles in close proximity to the plasma membrane (Fig. 1D-D″). The amount of Pkaap protein colocating with Rab11 vesicles was significantly reduced in *pkaap^RNAi^* larval fat body cells, when compared to control (*P*<0.0001; Fig. 1C). In addition, the morphology of Rab11 vesicles was altered, with the appearance of larger endosomes in the fat body tissue from *pkaap^RNAi^* (Fig. 1E,E″,F,F″), when compared to control larvae that exhibited smaller Rab11 vesicles (Fig. 1D,D″). The distribution and morphology of Rab11 vesicles was examined in relation to the plasma membrane, which was stained with CellMask™ Deep Red in live tissues. There were significantly more small ≤1 μm^2^ Rab11 vesicles (*P*<0.0001) near the plasma membrane of fat body cells in control larvae (Fig. 2A,A′) than for *pkaap^RNAi^* transgenic larvae (Fig. 2B,B′,C,C′,D). Moreover, there was a concomitant appearance of large Rab11 endosomes with intraluminal vesicles in the intracellular regions of fat body cells from *pkaap^RNAi^* transgenic larvae (Fig. 2B,B′,C,C′), when compared to control larvae (Fig. 2A,A′; Fig. S1A,A′). The size of these intracellular Rab11 endosomes was significantly increased in *pkaap^RNAi^*, when compared to control fat body cells (Fig. 2E). The Rab11 vesicle phenotype caused by *pkaap^RNAi^* (Fig. 2B,B′,C,C′) was similar to that observed for the GDP-bound dominant negative form of Rab11, *Rab11^S25N^* (Fig. 2D,E; Fig. S1C,C′), but not the GTP-bound constitutively active form of Rab11, *Rab11^Q70L^* (Fig. 2D,E; Fig. S1B,B′).

### The GTPase activities of Rab4 and Rab11 affected the intracellular distribution of Pkaap

**Fig. 3.**
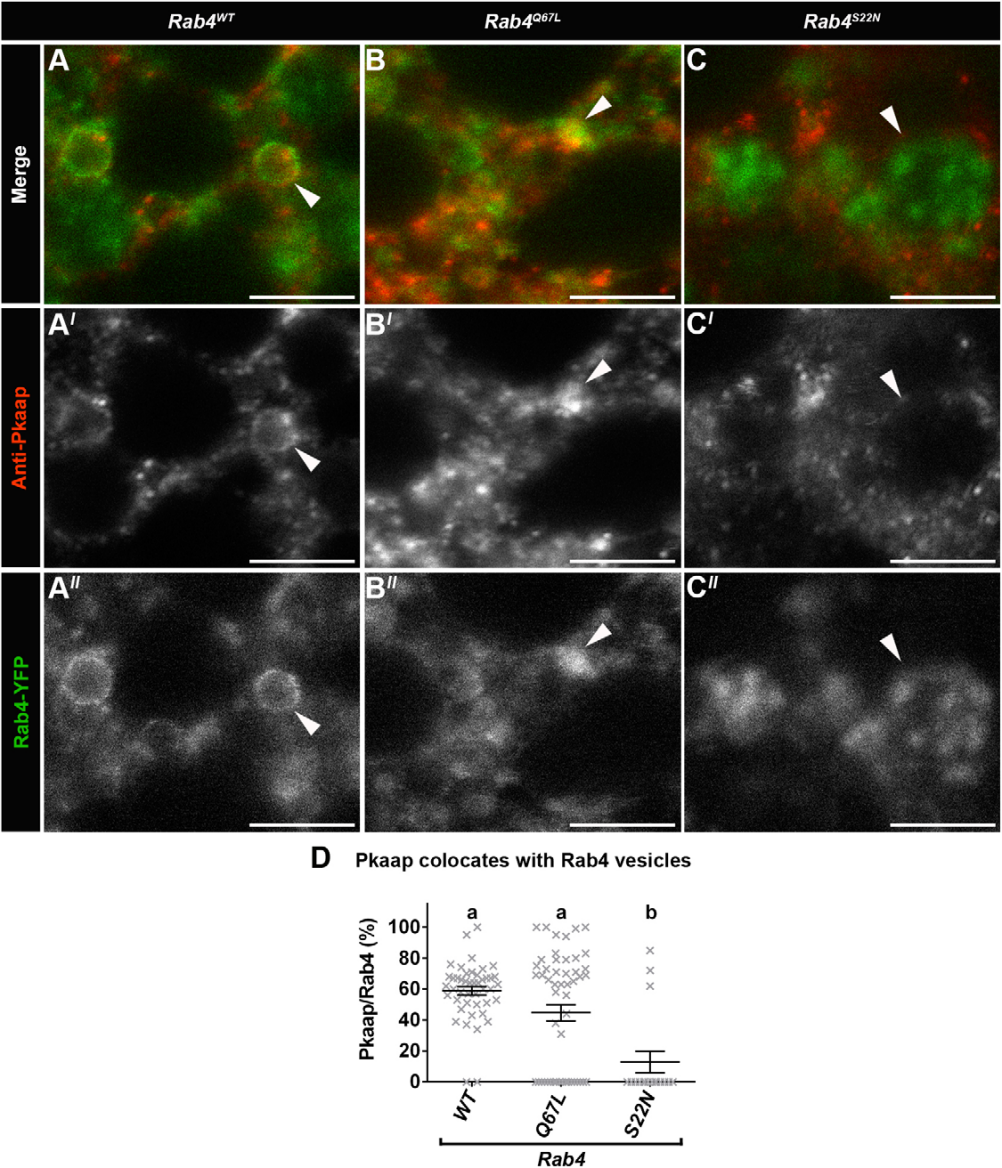
**Pkaap colocates with Rab4 endosomes.** (A-C) Confocal micrographs showing localisation of Pkaap detected with an anti-Pkaap antibody (red in A-C; greyscale in A′-C′) in relation to Rab4-YFP endosomes (green in A-C; greyscale in A″-C″) in fat body cells. Representative larval fat body cells were from the following genotypes: (A-A″; *Rab4^WT^*) wild-type control of Rab4, (B-B″; *Rab4^Q67L^*) GTP-bound constitutively active form of Rab4 and (C-C″; *Rab4^S22N^*) GDP-bound dominant negative form of Rab4. Scale bar: 5 μm. (D) Percentage of Pkaap colocation with Rab4-YFP vesicles in the fat body cells from *Rab4^WT^*, *Rab4^Q67L^* and *Rab4^S22N^*. One-way ANOVA and Tukey's multiple comparison test showed significant differences between the means for the genotypes (depicted by different letters on the bars, *P*<0.0001). Data presented as mean±s.e.m.

In fat body cells from larvae expressing wild-type Rab4 (*Rab4^WT^*), Pkaap was detected on Rab4-YFP vesicles (Fig. 3A-A″) and in the cytosol (as previously observed for D-AKAP2; Eggers et al., 2009). In fat body cells expressing constitutively active *Rab4^Q67L^*, while the size of the Rab4 vesicles was reduced, Pkaap was still detected colocating with Rab4 vesicles (Fig. 3B-B″). In contrast, in fat body cells expressing dominant negative *Rab4^S22N^*, the size of the Rab4 vesicles was increased and Pkaap showed no colocation with these vesicles, but rather was only detected in the same proximity as these Rab4 endosomes (Fig. 3C-C″). Quantitative analysis showed that the expression of *Rab4^S22N^* significantly reduced Pkaap association with Rab4 vesicles, when compared to *Rab4^WT^* and *Rab4^Q67L^* (*P*<0.0001; Fig. 3D).

**Fig. 4.**
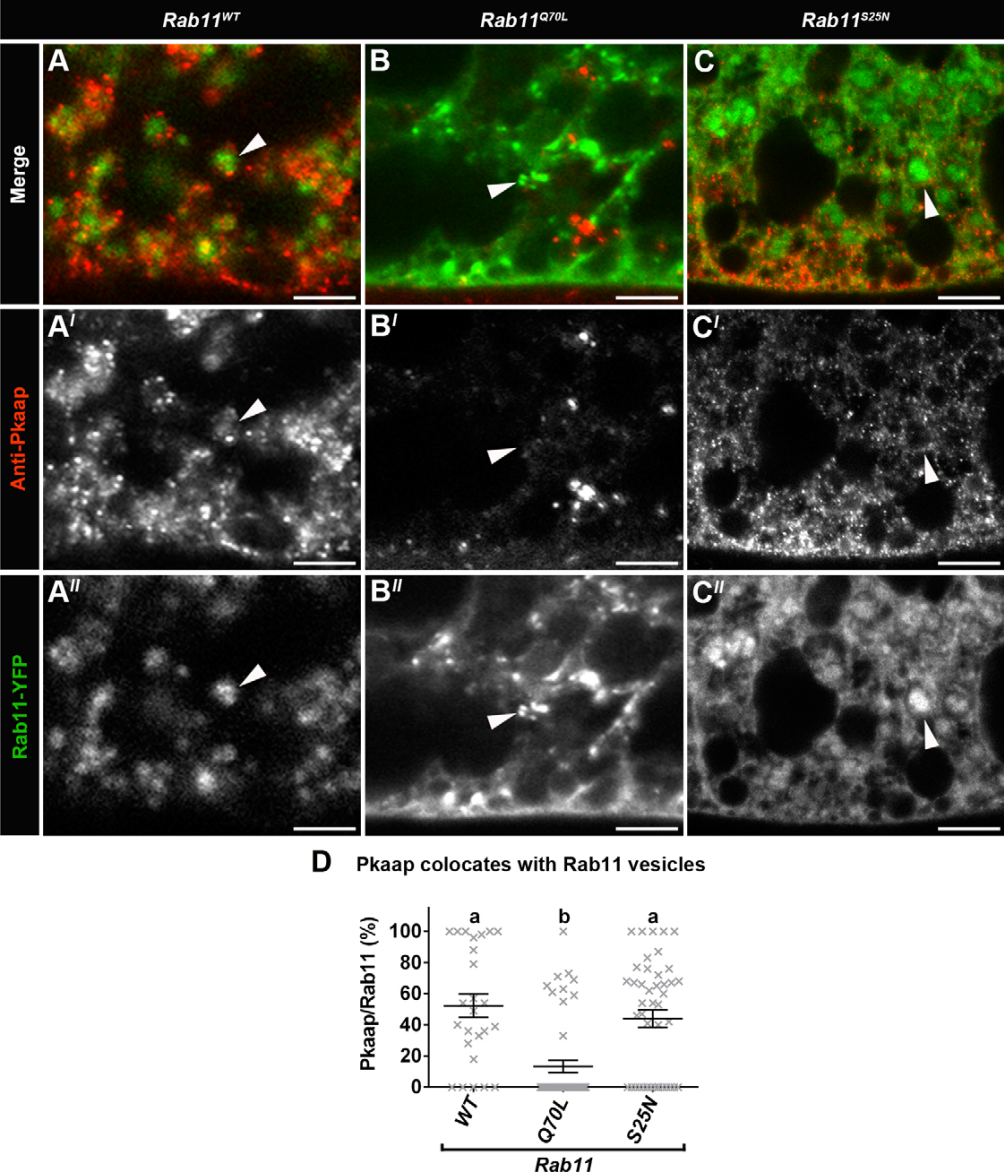
**Pkaap colocates with Rab11 endosomes.** (A-C) Confocal micrographs showing localisation of Pkaap detected with anti-Pkaap antibody (red in A-C; greyscale in A′-C′) in relation to Rab11-YFP endosomes (green in A-C; greyscale in A″-C″) in fat body cells. Representative larval fat body cells were from the following genotypes: (A-A″; *Rab11^WT^*) wild-type contol of Rab11, (B-B″; *Rab11^Q70L^*) GTP-bound constitutively active form of Rab11 and (C-C″; *Rab11^S25N^*) GDP-bound dominant negative form of Rab11. Scale bar: 5 μm. (D) Percentage of Pkaap colocation with Rab11-YFP vesicles in the fat body cells from *Rab11^WT^*, *Rab11^Q70L^*, and *Rab11^S25N^*. One-way ANOVA and Tukey's multiple comparison test showed significant differences between the means for the genotypes (depicted by different letters on the bars, *P*<0.0001). Data presented as mean±s.e.m.

In fat body cells from larvae expressing wild-type Rab11 (*Rab11^WT^*), Pkaap was detected in association with Rab11 endosomes (Fig. 4A-A″), and also showed diffused cytosolic localisation (as previously observed for D-AKAP2; Eggers et al., 2009). However, there was little or no Pkaap detected on Rab11 vesicles in fat body cells expressing constitutively active *Rab11^Q70L^* (Fig. 4B-B″). In fat body cells expressing dominant negative *Rab11^S25N^*, Pkaap showed significant interaction with Rab11 endosomes (Fig. 4C-C″), particularly in the vicinity of the plasma membrane. Quantification confirmed that *Rab11^WT^* and *Rab11^S25N^* resulted in Pkaap association with Rab11 endosomes, while there was significantly less Pkaap colocation with Rab11 endosomes for *Rab11^Q70L^* (*P*<0.0001; Fig. 4D).

### *pkaap* depletion resulted in altered Rab4/Rab11 recycling endosome morphology

**Fig. 5.**
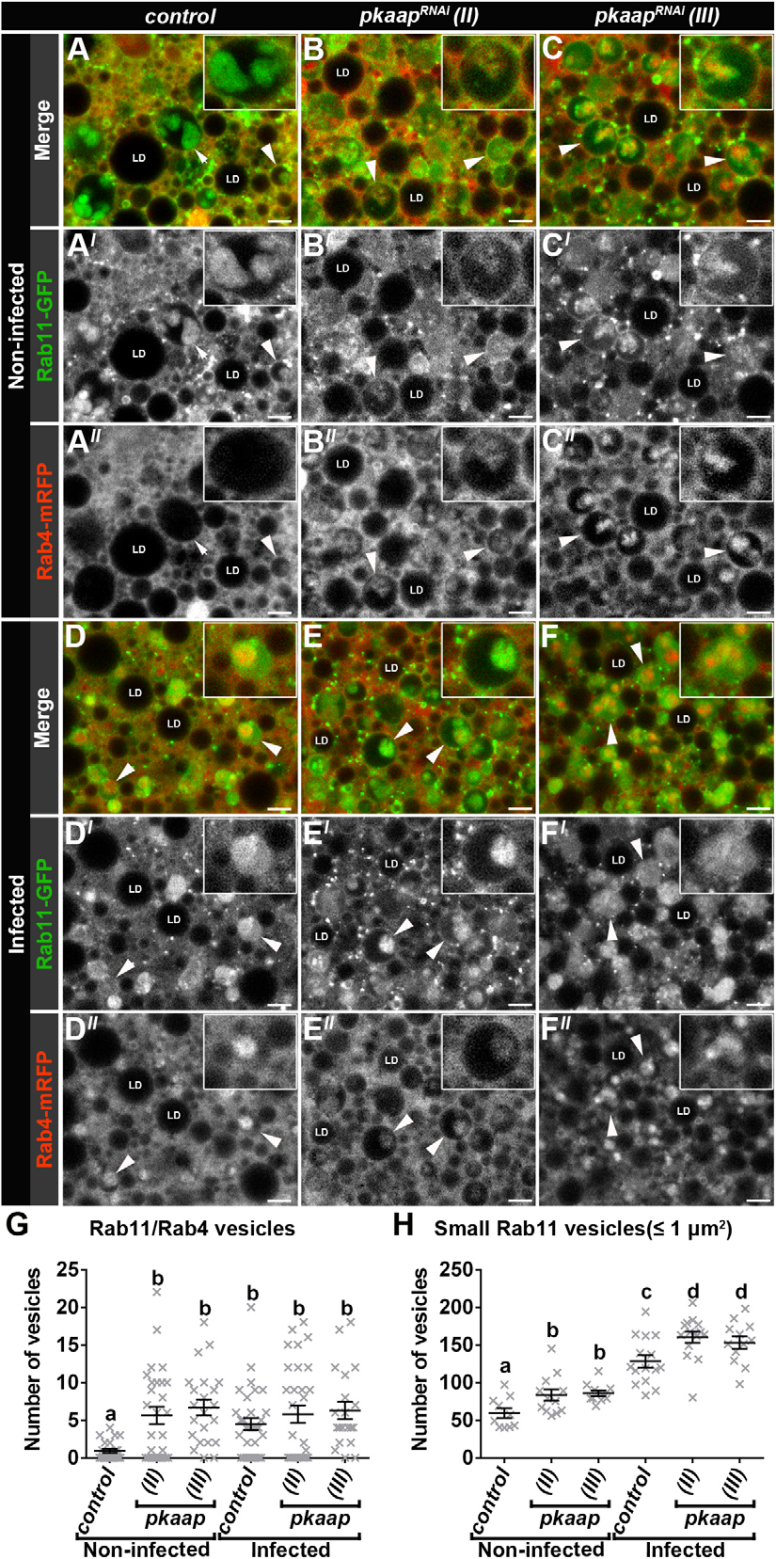
***pkaap* depletion altered the morphology of Rab4/Rab11 endosomes.** (A-F) Confocal micrographs of cross-section through the fat body cells showing Rab11-GFP (green in A-F; greyscale in A′-F′) and Rab4-mRFP vesicles (red in A-F; greyscale in A″-F″) in close proximity to the perinuclear region. Fat body cells were visualised from (A-C) non-infected larvae and (D-F) larvae infected orally with *Micrococcus luteus* for 4 h. Representative larval fat body cells were from the following genotypes: (A,D) control, (B,E) *pkaap^RNAi chromosome II^* and (C,F) *pkaap^RNAi chromosome III^*. Arrow depicts Rab11-GFP endosome (A). Arrowheads depict Rab4/Rab11 endosomes. LD, lipid droplets. Scale bar: 5 μm. (G) Histogram showing comparative analysis of the number of Rab4/Rab11 endosomes. One-way ANOVA and Tukey's multiple comparison test showed significant differences between the means for the genotypes (depicted by different letters on the bars, *P*=0.0025). (H) Histogram showing comparative analysis of the number of small ≤1 μm^2^ intracellular Rab11 vesicles. One-way ANOVA and Tukey's multiple comparison test showed significant differences between the means for the genotypes (depicted by different letters on the bars, *P*<0.0001). Data presented as mean±s.e.m.

In fat body cells from control larvae, there were large Rab11 endosomes that contained Rab11 intraluminal vesicles; as well as other smaller Rab11 vesicles (Fig. 5A). Recycling endosomes with Rab4 and Rab11 colocalisation were identified, but the larger Rab11 multivesicular endosomes did not appear to contain appreciable amounts of Rab4 (Fig. 5A-A″). In *pkaap^RNAi^* fat body cells, there were Rab4 compartments that contained little or no Rab11 (Fig. 5B-C″), and the larger Rab11 endosomes had intraluminal vesicles with both Rab4 and Rab11 (Fig. 5B-C″); with intraluminal Rab4/Rab11 vesicles in 64.6±8.3% for *pkaap^RNAi^* ^*chromosome II*^ and 70.9±8.9% for *pkaap^RNAi^* ^*chromosome III*^ compared to 13.1±7.2% in the controls. There was also an increase in the number of small Rab11 vesicles at the periphery of the larger multivesicular endosomes (Fig. 5B,B′,C,C′), particularly for *pkaap^RNAi^* ^*chromosome III*^ (Fig. 5C,C′).

The formation of Rab4/Rab11 multivesicular endosomes was also investigated under conditions of bacterial challenge to help define the role of Pkaap during an innate immune response. At four hours post-infection with *Micrococcus luteus* (Fig. 5D-F), there were variable amounts of Rab4 (e.g. Fig. 5E,E″), while there were similar numbers of Rab4/Rab11 multivesicular endosomes in the fat body cells from control and *pkaap^RNAi^* larvae; with 65.5±7.9% in control and 70.4±8.4% for *pkaap^RNAi^* ^*chromosome II*^, 77.3±6.7% for *pkaap^RNAi^* ^*chromosome III*^. Consequently, while there was a significant increase in the number of Rab4/Rab11 multivesicular endosomes in infected control cells, there was not a significant difference between the number of Rab4/Rab11 multivesicular endosomes before and after infection in *pkaap^RNAi^* fat body cells (*P*=0.0025; Fig. 5G). In control cells there were also significantly more small Rab11 vesicles (≤1 μm^2^) after infection (*P*<0.0001; Fig. 5D,D′,H), and more small Rab11 vesicles in *pkaap^RNAi^* fat body cells (*P*<0.0001; Fig. 5E,E′,F,F′,H).

### *pkaap* depletion resulted in reduced antimicrobial peptide Drosomycin delivery to the plasma membrane

**Fig. 6.**
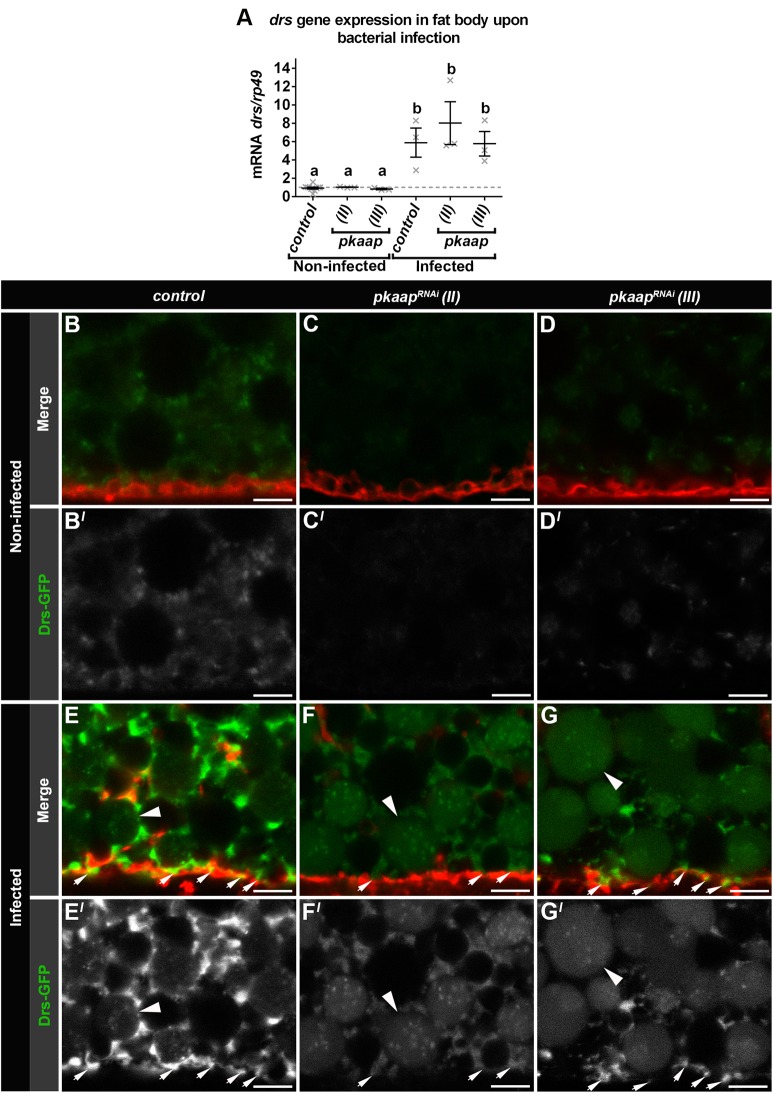
***pkaap* depletion reduced the amount of Drosomycin at the plasma membrane.** (A) The expression of *drosomycin* (*drs*) mRNA was characterised by quantitative real-time PCR. mRNA levels were normalised against *rp49* mRNA levels. Three independent sets of samples were collected from late third larval instar (−4 h puparium formation) from non-infected larvae and larvae infected orally with *Micrococcus luteus* and *Escherichia coli*. One-way ANOVA and Tukey's multiple comparison test showed significant differences between the means in genotypes (depicted by different letters on the bars, *P*<0.0001). Data presented as mean±s.e.m. (B-G) Confocal micrographs showing the intracellular distribution of Drosomycin-GFP in fat body cells (green in A-G; greyscale in A′-G′) with the plasma membrane outlined by CellMask™ Deep Red (red in A-G). Fat body cells were visualised from (B-D) non-infected larvae and (E-G) larvae infected orally with *Micrococcus luteus* for 4 h. Arrows depict Drosomycin-GFP at the plasma membrane. Arrowheads depict large Drosomycin-GFP compartments. Representative larval fat body cells were from the following genotypes: (B,E) control, (C,F) pkaap^RNAi chromosome II^ and (D,G) pkaap^RNAi chromosome III^. Scale bar: 5 μm.

To determine the effect of *pkaap^RNAi^* on innate immune cargo delivery, we examined by quantitative real-time PCR the expression of the antimicrobial peptide Drosomycin in fat body cells, either before or after infection. In non-infected control larvae, only minimal Drosomycin was detected prior to infection (Fig. 6A for mRNA and protein visualised in Fig. 6B,B′), but there was a marked increase in Drosomycin expression four hours after infection (Fig. 6A for mRNA and protein visualised in Fig. 6E,E′). In addition, the *Drosophila* NF-κB homolog Dorsal, which normally activates Drosomycin transcription in response to infection (Manfruelli et al., 1999), was translocated from the cytoplasm to the nucleus in *pkaap^RNAi^* transgenic larvae, and there were no differences in Dorsal subcellular localisation for control and *pkaap^RNAi^* [i.e. Dorsal was cytoplasmic in non-infected larvae (Fig. S2A-C) and predominantly nuclear within 30-60 min after infection (Fig. S2D-I)]. In *Micrococcus luteus*-infected control larvae (Fig. 6E,E′), the Drosomycin-GFP cargo was detected in small ∼1 µm^2^ vesicles either in close proximity to larger vesicles that contained less concentrated cargo or near the cell surface colocating with CellMask™ Deep Red. In contrast, for *pkaap^RNAi^* larvae there was little or no detectable Drosomycin-GFP protein detected prior to infection (Fig. 6C-D′), even though the level of mRNA was similar to that detected in control fat body tissue (Fig. 6A). While there was an increase in mRNA expression in response to infection in *pkaap^RNAi^* fat body cells (Fig. 6A), there was only a relatively small amount of Drosomycin-GFP detected, when compared to control larvae (Fig. 6F-G′). This Drosomycin-GFP, in *pkaap^RNAi^* fat body cells, was detected as a diffuse signal in large vesicular structures that contained intraluminal vesicles with only minimal Drosomycin-GFP (Fig. 6F-G′). In addition, there was a reduced amount of this Drosomycin-GFP cargo near the cell surface (Fig. 6F-G′), when compared to controls (Fig. 6E,E′).

## DISCUSSION

Following host invasion, cells of the innate immune system detect microbial pathogens via pattern recognition receptors and peptidoglycan-recognition receptors (Ferrandon et al., 2007; Royet et al., 2005). These receptors can trigger the activation of immune cells by two distinct intracellular cascades; the Toll-interleukin 1 pathway and the immune deficiency-TNFα pathway (Lemaitre, 2004). This innate immune signalling initiates a sequence of events that leads to the transcription of pro-inflammatory cytokine and antimicrobial peptide genes (Beisswenger and Bals, 2005; Cederlund et al., 2011; Jenssen et al., 2006; Lai and Gallo, 2009; Medzhitov, 2008; Stow and Murray, 2013). Newly synthesised immune-mediators are transported through the endoplasmic reticulum–Golgi network and delivered to endosomes for final processing and packaging into Rab11 vesicles; ready for exocytosis at the plasma membrane. Endosomal trafficking is regulated by a complex set of vesicular machinery including, for example A-kinase anchoring proteins, and here, we have investigated the role of the *Drosophila* A-kinase anchoring protein, Pkaap, in the control of innate immune secretion.

Relatively little is known about how A-kinase anchoring proteins regulate immune defence mechanisms, although they have been previously implicated in modulating the transcriptional activation of cytokines in immune cells (Kim et al., 2011; Shibolet et al., 2007; Wall et al., 2009; Williams, 2002). For instance, AKAP13 has a role in the nuclear translocation of transcription factor NF-κβ, and therefore the TLR2-dependent activation of pro-inflammatory cytokine secretion (Shibolet et al., 2007). In contrast, AKAP95 was required for targeting of type II regulatory subunit of PKA to supress the early expression of *TNFα* in lipopolysaccharide-stimulated RAW264.7 macrophages (Wall et al., 2009). D-AKAP2-anchored type I regulatory subunit of PKA is involved in prostaglandin E2 potentiation of lipopolysaccharide-induced nitric oxide synthesis downstream of Toll-like receptors and the expression of interleukin 6 and interleukin 10 in alveolar macrophages (Kim et al., 2011). Our analysis of *pkaap* depleted larvae showed a normal activation of immune response pathways, resulting in Dorsal nuclear translocation and antimicrobial peptide *drosomycin* gene expression, suggesting that Pkaap did not have a role in modulating antimicrobial peptide transcriptional regulation. In future studies the effect of *pkaap^RNAi^* on phagocytosis and morphology of Rab4/Rab11 endosomes in haemocytes might be determined as the expression of transgenes was driven by fat body- and haemocyte-specific *CG-GAL4* driver. Given that D-AKAP2 is involved in the regulation of transferrin receptor recycling via Rab4/Rab11 endosomes (Eggers et al., 2009) and there were no difference in Rab11-GFP fluorescence in control and *pkaap^RNAi^* fat body cells, it was postulated that Pkaap might be involved in antimicrobial peptide intracellular traffic.

In *Drosophila*, antimicrobial peptide targeting to the plasma membrane is known to involve Rab4 and Rab11 GTPases (Shandala et al., 2011). GAPs and GEFs control membrane trafficking by modulating Rab protein activities (Bos et al., 2007; Csépányi-Kömi et al., 2012; Liao et al., 2008); and the nucleotide-dependent location of Pkaap to endosomes suggested that it could have a role as both a GAP and GEF. We found that Pkaap colocated with wild-type and GTP-bound constitutively active Rab4, but not the GDP-bound dominant negative form of Rab4, which was consistent with it acting as a GAP for Rab4. We also showed that Pkaap colocated with wild-type and GDP-bound dominant negative form of Rab11, but not GTP-bound constitutively active Rab11, suggesting that it may also be acting as a GEF for Rab11. This was consistent with observations of D-AKAP2 co-immunoprecipitation studies for both Rab4 and Rab11 GTPases in HEK293 cells (Eggers et al., 2009; Fukuda et al., 2008) and that some AKAPs have been shown to act as molecular switches in regulating GTPase activity. For example, AKAP-Lbc binds to the GDP-bound or nucleotide-free forms of RhoA and possesses Rho-selective GEF activity in HeLa and HEK-293 cells (Diviani et al., 2004, 2001). Thus, by regulating the activity of Rab4 and Rab11, Pkaap could modulate two critical steps during the vesicular traffic of cargo along the immune secretory pathway, cargo sorting in recycling endosomes and exocytosis at the plasma membrane.

The depletion of *pkaap* had a direct effect on Rab11 vesicle traffic and antimicrobial peptide immune cargo delivery to the plasma membrane and resulted in the accumulation of enlarged Rab11 vesicles at the cellular periphery. The GDP-bound dominant negative form of Rab11 also reduced the number of small Rab11 vesicles in close proximity to the cell surface and resulted in the accumulation of enlarged Rab11 multivesicular endosomes, suggesting that *pkaap* depletion trapped Rab11 in a GDP-bound form. These enlarged Rab11 compartments with intraluminal vesicles were identified as multivesicular endosomes by definition. To further explore this question, future work may investigate fat body tissues expressing Rab11-GFP with antibodies detecting other multivesicular endosome markers, such as Hrs or Vps16. Constitutively active Rab11 produced a different vesicular phenotype, with enhanced delivery of small Rab11 vesicles to the plasma membrane. The results also revealed low Pkaap levels in fat body cells expressing constitutively active form of Rab11, suggesting a negative feedback mechanism by which the intracellular level of GEF proteins is regulated, but this is yet to be determined. Immune cargo delivery is dependent on Rab11 vesicles (Shandala et al., 2011) and *pkaap* depletion abrogated the intracellular traffic and delivery of the antimicrobial peptide Drosomycin to the plasma membrane upon bacterial challenge, but did not cause changes in the size of the Rab4/Rab11 endosomes. Concomitantly, *pkaap* depletion caused Drosomycin to accumulate in Rab4/Rab11 endosomes and this reduction in antimicrobial peptide delivery coupled with reduced secretion into the haemolymph could explain reduced larval viability following bacterial challenge. Due to limitations in the availability of transgenic stocks that could be used to study secreted proteins in live mode, we were limited to using Drosomycin-GFP and ideally the findings should be confirmed with other immune mediators. It would also be interesting to determine whether treatment of *pkaap^RNAi^* transgenic larvae with beta-lactam antibiotics (e.g. tetracycline) can improve survival rates after infection with *Micrococcus luteus*.

The structural changes to Rab4/Rab11 endosomes caused by *pkaap* depletion could affect the sorting and compartmentalisation of immune cargo in recycling endosomes. This would be consistent with the effects that *pkaap* depletion had on both of the GTPases Rab4 and Rab11 and the morphology of Rab4/Rab11 endosomes. Instead of correct antimicrobial peptide cargo sorting in Rab4/Rab11 recycling endosomes and packaging into Rab11 vesicles, a large amount of Drosomycin appeared to be degraded. For example, after immune challenge, while *drosomycin* mRNA expression was not reduced in response to *pkaap* depletion there was less Drosomycin protein, suggesting that a lysosomal degradative pathway may have been evoked in response to failed sorting in Rab4/Rab11 recycling endosomes; therapeutic agents, such as bafilomycin A and chloroquine, could be used in future studies. The specific effects of *pkaap* depletion on the dynamics and morphology of different endosomes suggested that *pkaap^RNAi^* transgenic larvae could have multiple impairments in the antimicrobial peptide trafficking and secretion pathway that contributed to immune dysfunction.

**Fig. 7.**
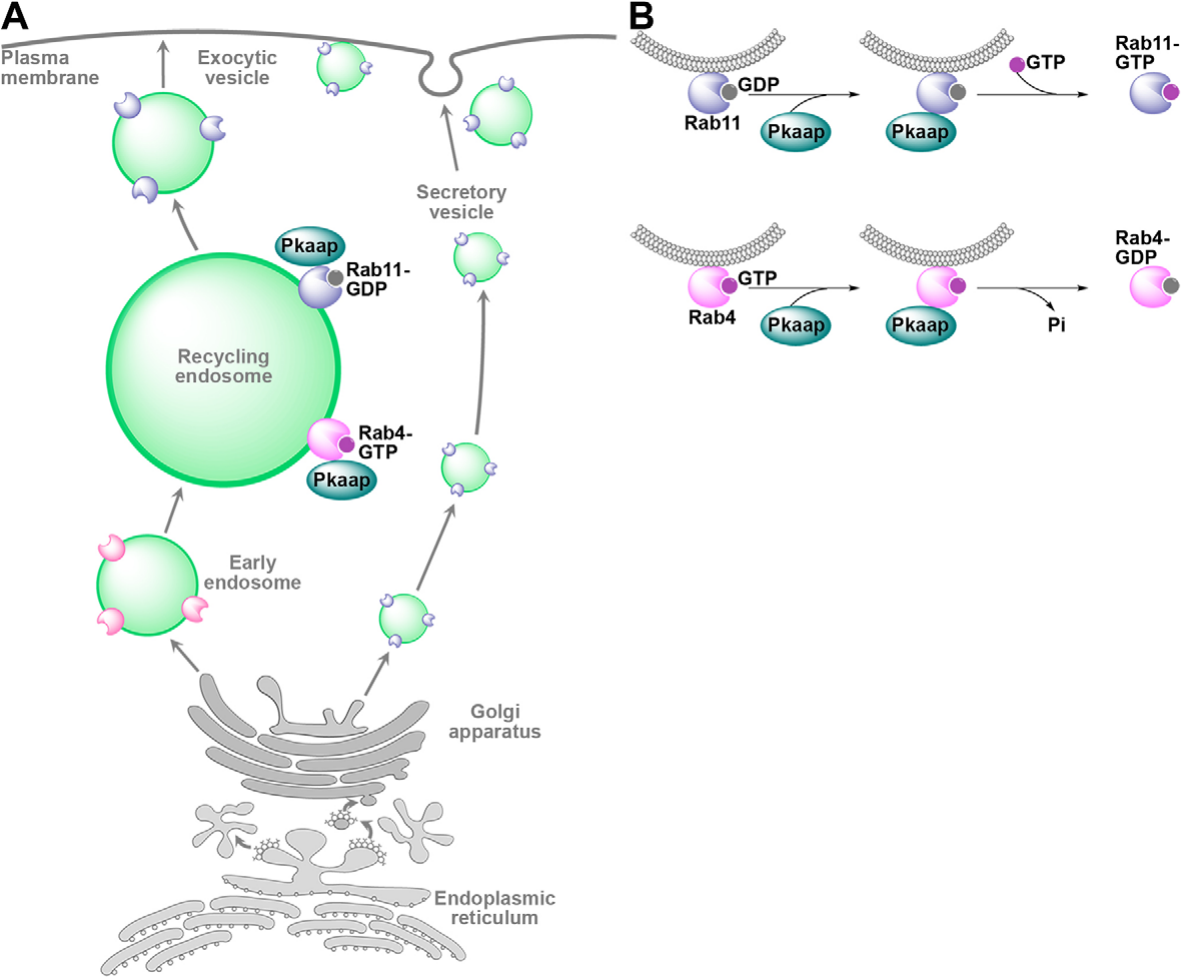
**The role of Pkaap in the regulation of antimicrobial peptide exocytosis in fat body cells.** (A) Schematic representation of the nucleotide-dependent location of Pkaap to recycling endosomes. Pkaap binds to GTP-bound constitutively active form of Rab4 and GDP-bound dominant negative form of Rab11. (B) Schematic diagram showing the role of Pkaap in the regulation of membrane trafficking by modulating Rab4 and Rab11 activities. GTP, guanosine triphosphate; GDP, guanosine diphosphate.

We concluded that Pkaap has a critical role in effecting an innate immune response and is important for *Drosophila* viability. The colocation of Pkaap with Rab11 endosomes suggested that Pkaap might be acting as a regulator of exocytosis and the effect of *pkaap* depletion on Rab11 vesicle and antimicrobial peptide cargo delivery would support this hypothesis (Fig. 7A,B). However, Pkaap also appeared to have an important role in Rab4/Rab11 recycling endosome morphology and function suggesting that it is also involved in cargo sorting and delivery earlier in the secretory pathway. It appears that Pkaap has a dual role in antimicrobial peptide trafficking and exocytosis making it an essential component for the secretion of inflammatory mediators and the defence of the host against pathogens. In future studies, the role of Pkaap might be investigated in other secretory tissues, such as the salivary glands, to ascertain if a similar molecular mechanism is used to control non-immune secretion.

## MATERIALS AND METHODS

### Fly stocks

Fly stocks were maintained in standard medium at 25°C. The yeast *GAL4-UAS* two component system was used for fat body-specific expression of genes of interest (Brand and Perrimon, 1993; Schmid et al., 2014). Expression of transgenes from the *UAS* was driven by *CG-GAL4* (Asha et al., 2003). RNA interference (RNAi) silencing stocks of *pkaap*, *UAS-pkaap^RNAi^* ^*chromosome II*^ and *UAS-pkaap^RNAi^* ^*chromosome III*^, were obtained from the Vienna *Drosophila* RNAi Centre (Vienna, Austria; Dietzl et al., 2007). Transgenic stocks *UAS-Rab4^WT^-YFP*, *UAS-Rab4^Q67L^-YFP*, *UAS-Rab4^S22N^-YFP*, *UAS-Rab11^WT^-YFP*, *UAS-Rab11^Q70L^-YFP* and *UAS-Rab11^S25N^-YFP* were obtained from the Bloomington *Drosophila* Stock Center (Indiana University, IN, USA; Zhang et al., 2007). Transgenic stocks *UAS-Rab4-mRFP* and *UAS-Rab11-GFP* were obtained from Markos González-Gaitán (University of Geneva, Geneva, Switzerland; Entchev et al., 2000; Wucherpfennig et al., 2003) and Donald F. Ready (Purdue University, West Lafayette, IN; Satoh et al*.*, 2005). *Drosomycin-GFP* was a kind gift from Dominique Ferrandon (Equipe Fondation Recherche Médicale, Strasbourg, France; Ferrandon, 2007).

### Natural bacterial infection

*Drosophila* early third larval instars were infected by the oral route with *Micrococcus luteus* and *Escherichia coli* for four hours at 25°C (to avoid temperature stress; Shandala et al., 2011). Fat body tissues were collected and stored at −80°C until required for antimicrobial peptide *drosomycin* gene expression analysis by quantitative real-time PCR. Secretion of the antimicrobial peptide Drosomycin into the haemolymph was not assessed because insect hemolymph had variable degrees of clotting (and therefore caused issues for Drosomycin recovery), making this task technically impossible. To assess survival rates (Shandala et al., 2011), pupae were scored as either live or dead within one week following infection and for each group (control, *pkaap^RNAi chromosome II^* and *pkaap^RNAi chromosome III^*) n=1200-1400 individual larvae were scored. The genotypes used for these experiments were *CG-CAL4>+/+; +/+* (control), *CG-CAL4>UAS-pkaap^RNAi^/+; +/+* (*pkaap^RNAi chromosome II^*) and *CG-CAL4>+/+; UAS-pkaap^RNAi^/+* (*pkaap^RNAi chromosome III^*).

### Gene expression

For quantitative real-time PCR analysis, RNA was isolated from the fat body tissue of 30 larvae using an RNAqueous^®^ kit according to the manufacturer's protocol (Ambion, USA). cDNA was synthesised using a High Capacity RNA-to-cDNA kit (Applied Biosystems, USA). Quantitative real-time PCR was performed using a 7500 Fast Real-Time PCR System (Applied Biosystems, USA) using Fast SYBR^®^ Green Master Mix kit (Applied Biosystems, USA). Three independent biological samples were analysed for each genotype. The mRNA expression of genes was normalised against the endogenous control gene *rp49*, using the ΔΔCT method. PCR primers were obtained from GeneWorks (Adelaide, Australia). The primers used for the quantitative real-time PCR were: *pkaap* (CG4132) forward, 5′-CTCCGATGGCATCAGTCTCG-3′, and reverse, 5′-CAGGCAGTTCGGATCGTTGA-3′; *drosomycin* (CG10810) forward, 5′-GTACTTGTTCGCCCTCTTCG-3′, and reverse, 5′-ATTTAGCATCCTTCGCACCA-3′; and *rp49* (CG7939, used as an endogenous control) forward, 5′-CGAGTTGAACTGCCTTCAAGATGACCA-3′, reverse 5′-GCTTGGTGCGCTTCTTCACGATCT-3′.

### Live cell imaging

For *ex vivo* live cell imaging, the fat body tissues were dissected from late third larval instars (−4 h before puparium formation) and incubated with CellMask™ Deep Red Plasma Membrane Stain (Applied Biosystems, USA) for 2 min at room temperature. Fat body tissues were attached to a coverslip using Carbomer 940-based gel (Snowdrift Farm, USA) and analysed for a maximum of 30 min; as a slower motility for endosome vesicles was observed after this time period (Sorvina et al., 2013).

### Immunostaining and confocal microscopy

Fat body tissues expressing simultaneously Rab4-mRFP/Rab4-YFP and Rab11-GFP/Rab11-YFP were imaged in order to show the similarity in the distribution of the endosomes in these transgenic lines (Fig. S3A,B). *Drosophila* fat body tissues were fixed and stained as previously described (Shandala et al., 2011). Antibodies used for immunofluorescence were rabbit polyclonal anti-Pkaap (GeneScript, USA) and mouse monoclonal anti-Dorsal 7A4 (DSHB, USA). Anti-Pkaap antibody was pre-absorbed against fixed fat body tissues in 5% bovine serum albumin for 45 min at room temperature. This custom-made anti-Pkaap antibody only worked for immunofluorescence and did not work for western blot analysis or co-immunoprecipitation, and therefore protein-protein interaction studies were not technically possible due to problems with current reagents. Secondary anti-IgG antibody conjugates with Cy3 and Cy5 labels were obtained from Jackson Immuno Research Laboratories (Shandala et al., 2011). The Alexa Fluor^®^ 488 Phalloidin, Alexa Fluor^®^ 568 Phalloidin and Hoechst 33258 DNA stain were obtained from Invitrogen, USA. Imaging was performed using a Zeiss LSM710 NLO confocal microscope equipped with argon-gas, 543 nm and 633 nm solid-state lasers (Zeiss, Germany) and a two-photon Mai-Tai^®^, tunable Ti:Sapphire femtosecond pulse laser (Spectra-Physics, USA). All images were acquired using a Plan-APOCHROMAT 63×/NA 1.4 oil immersion objective to enable detailed visualisation of endosome/exocytic compartments. Each confocal micrograph represented 1.5 μm thin optical sections and the data was representative of at least ten independent replicates.

### Image processing

The final preparation of the figures was conducted with Adobe Photoshop CS6 (Adobe Systems Inc.).

### Measurements and statistical analysis

The number of small ≤1 µm^2^ Rab11 vesicles at the plasma membrane (22 µm sections), the size of intracellular Rab11 endosomes within a total cells area, and the number of Rab4/Rab11 recycling endosomes within 60 µm^2^ regions of interest was defined using Volocity 6.2.1 software.

The difference between group means was assessed by one-way analysis of variance (ANOVA), with individual group variance assessed by a Bartlett's test. Where the level of significance was *P*<0.05, post-hoc tests were performed using a Tukey's multiple comparison test (using GraphPad Prism version 6.00 for Windows, GraphPad Software, San Diego, CA USA). Data was presented as the mean±s.e.m.
